# Biodiversity thresholds in invertebrate communities: The responses of dung beetle subgroups to forest loss

**DOI:** 10.1371/journal.pone.0201368

**Published:** 2018-08-10

**Authors:** Clarissa Machado Pinto Leite, Eduardo Mariano-Neto, Pedro Luís Bernardo da Rocha

**Affiliations:** Programa de Pós-Graduação em Ecologia e Biomonitoramento, Instituto de Biologia, Universidade Federal da Bahia, BA, Brazil; Sichuan University, CHINA

## Abstract

Extinction thresholds have been predicted to be critical values of habitat loss in which an abrupt reduction in populations occurs through the interaction between reduced habitat and increased isolation in the landscape. In communities, extinction thresholds are referred to as ‘biodiversity thresholds’. The biodiversity threshold values documented so far occur between 30% and 50% of habitat cover in landscapes. However, the assessment of biodiversity thresholds has mainly focused on vertebrate and plant communities. Here, we evaluated the occurrence of biodiversity thresholds in dung beetle communities by sampling ten 3,600 ha Atlantic Forest landscapes with forest cover ranging from 5% to 55%. We analysed the response patterns (abundance, gamma and mean alpha diversity) of community subgroups with different levels of forest dependency (forest species, generalist species, and open-area species) using model selection, comparing null, linear, bell-shaped and logistic models. The response of the community of forest species equally fits both linear and logistic models predicting a biodiversity threshold at 25% forest cover. Generalist species showed peak abundance at 20% forest cover although this result reflects a very poor generalist assembly. Open-area specialists did not respond to the amount of forest. The two most plausible models for forest species suggest two different biodiversity management options. Since the biodiversity threshold model represents a more dramatic scenario for the loss of biodiversity in Atlantic forest landscapes, we suggest, based on precautionary principle, that our results should strength guidelines that consider minimum values of forest cover in management strategies to avoid abrupt biodiversity loss and impacts on ecosystem services.

## Introduction

Over the past few years, habitat loss has repeatedly been cited as the principal driver of the current biodiversity crisis [1–3]. As a result, many authors have documented the effects of habitat loss on different biological groups using different measures of biodiversity [4–14]. Over the course of habitat loss in the landscape, there are some changes in the landscape configuration, such as the number and size of patches, the distance between them, and the quality of patches (e.g., increasing edge areas). These changes influence the persistence of natural populations and invasion by foreign species [15,16]. Therefore, ‘extinction thresholds’ were predicted to be critical values of habitat loss, in which a sharp reduction in populations occurs through the interaction between reduced habitat and increased isolation in the landscape [16]. This sharp reduction would occur due to reductions in resources and migration among the original habitat patches, which reduces the viability of populations and recolonisation after local extinctions [13]. Intending to simplify the view of the relationship between habitat distribution and species richness and emphasise the importance of the amount of habitat at the landscape scale, Fahrig proposed ‘the habitat amount hypothesis’ [17]. According to her proposal, a single predictor variable of species richness, i.e., habitat amount, could replace other variables related to the landscape configuration, such as patch size and fragmentation.

Some authors extended the idea of extinction thresholds to communities. In this case, a set of different species with similar responses to habitat loss could also become extinct at a particular amount of habitat or cause extinctions due to cascade effects in relationship networks. This phenomenon is referred to by some authors as a ‘biodiversity threshold’ [6,18]. With the development of studies and discussion on extinction thresholds, it is becoming increasingly clear that species with different degrees of specialisation to the original habitat should respond differently to habitat loss [19–21]. If different species were idiosyncratic in their response to habitat loss, suffering an abrupt loss in their densities in different parts of the gradient, it would not be possible to detect a threshold for the whole community.

However, a community associated with a particular habitat may include generalist species and specialist species. The former group of species would benefit by increased heterogeneity at intermediate levels of habitat cover since a maximum number of patches is expected to occur at approximately 30% of remaining habitat in the landscape [16]. The latter group of species would be affected by the loss of the original habitat and could become extinct at a particular habitat amount, since at approximately 60% of the original habitat in the landscape, there is a very strong decline in the maximum patch size, and there is a sharp increase in the average distance between patches at approximately 20% of forest cover [5,16]. Finally, in a landscape in which the original habitat is fragmented, alternative altered habitats can be invaded by new habitat specialists. They should be influenced by changes in the amount of original habitat in an inverse way when compared to specialists of the original habitat and may cause an adverse impact on native species. These patterns have been observed in empirical studies in different communities [5–7,9,11,12]. For example, forest specialist small mammal, birds and trees in Atlantic Forest tend to disappear abruptly in landscapes with forest cover that ranges from 30% to 50% [5–7,9,11,22]. Medium-sized mammals and birds in the Amazon disappear at less than 40% [12]. On the other hand, the abundance and richness of generalist small mammals and birds in Atlantic Forest are favoured by an increase in landscape structural heterogeneity at intermediate levels of forest cover (30% and 50%, respectively) [6,11].

Despite the growing number of empirical studies, the assessment of biodiversity thresholds has mainly focused on vertebrates and plant species. As far as we know, few large-landscape studies using invertebrates have been published [10,23], even considering their particular relevance in terms of ecosystem functioning and biodiversity conservation research [24]. Insect species may suffer from habitat loss in large landscapes not always because habitat remnants are too small to harbour viable population numbers, but due to loss of microclimate conditions as a result of edge effects [25,26] or through alterations to the composition or availability of resources provided by other locally extinct species due to cascade effects [27,28].

Dung beetles are stenotopic in terms of vegetation structure, as well as several related factors, such as atmospheric temperature, relative humidity, soil surface temperature and the degree of direct solar incidence [29]. In Neotropical forests, dung beetles have found ideal conditions under which to survive and diversify, with essential elements they need to feed and reproduce: mammalian faeces, carrion and rotten fruits [29]. It is well known that through the processing of such materials, dung beetles contribute to nutrient cycling, soil aeration, secondary seed burial and parasite suppression [30]. Through their usual dependence on mammalian faeces for nesting, dung beetles should be even more susceptible to the effects of forest loss, through the direct influence of this disturbance on mammals [5,6,12] and by increased hunting pressure as a result of the easier access to fragmented forests [27,28]. Considering that sharp losses in dung beetle diversity might affect the provisioning of some relevant ecosystem services [31,32], it is advisable to investigate the occurrence of thresholds in those communities. Furthermore, the use of a less common taxonomic group may provide insights about other types of communities (e.g., those with a smaller average size that explore the lower stratum of forests and are responsible for other ecological processes), broadening our focus and allowing more general conclusions about threshold models.

In regard to other communities, a preponderance of patch-scale studies have emphasised the deleterious effects of forest loss on dung beetle communities in tropical forests [25,33–41]. Factors such as patch size [33,37–39], patch isolation [37–39], edge effects [40], and matrix quality [38] show strong impacts on dung beetle communities. The robust and negative responses of tropical dung beetle communities to the increasing modification of tropical forests and declining fragment size were explored and supported by a meta-analysis in 2007 [38], and subsequent studies have increased the pessimistic expectations about the effects of tropical forest fragmentation on this group [37,39–42]. The different responses of community subgroups due to fragmentation was also observed in dung beetle community studies [37–41]. Edge-dominated and matrix habitats ensure the persistence of disturbance-adapted species while promoting the local extirpation of forest-dependent species in a fragmented landscape of Atlantic Forest remnants surrounded by sugar cane plantation [41]. Studies in the Amazon did not find evidence of density compensation by disturbance-adapted species when primary forests are replaced by tree plantations and secondary forests [42]. However, the replacement of forest with cattle pasture in proximity with savannas, such as the *Caatinga* or *Cerrado* biomes, and sandy areas near the coast known as *Restinga* [43] could promote less dung beetle density loss due to the invasion of open-area specialist species coming from those areas [44].

Finally, as far as we know, although several studies have measured forest cover at a landscape scale [14,39–41], only one has assessed changes in dung beetle community structure using landscapes as sample unity [14]. In that study, although the authors did not evaluate the occurrence of a biodiversity threshold, they found that a combination of patch size, landscape forest cover, and matrix composition were the best predictors of beetle abundance, richness and biomass in fragmented landscapes in Mexico [14].

Our primary aim was to evaluate the occurrence of biodiversity thresholds in dung beetle communities. We seek to answer how a reduction in Atlantic forest cover at the landscape scale modifies the abundance and diversity of community subgroups with different levels of forest habitat dependency. We tested three hypotheses (i) forest specialist dung beetles tend to disappear abruptly in landscapes with forest cover less than 30%, (ii) generalist dung beetles benefit from fragmentation at the landscape scale, showing a peak in the abundance and diversity of generalist species at intermediate levels of forest cover at the landscape scale, and (3) open-area specialist dung beetles will benefit from the loss of forested habitat at the landscape scale and become more abundant and diverse in landscapes with the lowest amount of forest cover.

## Materials and methods

This study was part of a project aimed to evaluate, based on a single sampling design, the effects of forest loss on different components of biodiversity [6,7,9,10].

### Ethics statement

Trapping, handling and specimens collections were approved by IBAMA—Instituto Brasileiro do Meio Ambiente e dos Recursos Naturais Renováveis (license number 12023–3). All specimens were collected only in private lands and all owners of the land gave us permission to conduct the study on their site. The specimens collected are deposited at Museum of Zoology, Universidade Federal do Mato Grosso, in compliance with national laws (IN 154/IBAMA). We did not sample either protected areas or species.

### Study area

**Fig 1:**
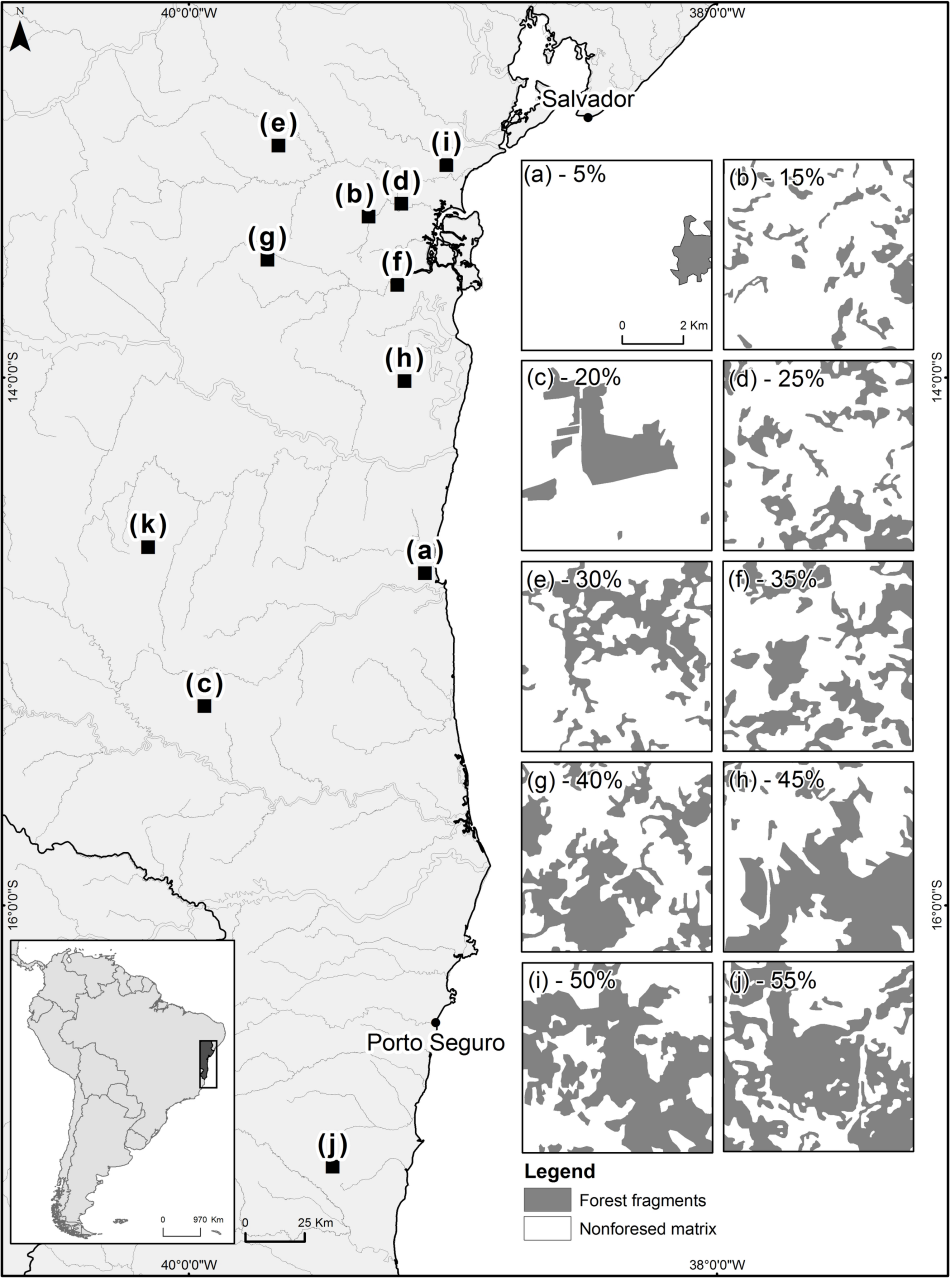
Map of the study area in the state of Bahia, north-eastern Brazil. In detail, at the bottom left, a map of Brazil indicating the region of the study area. The large image on the left side shows the geographical positions of the landscapes sampled along the eastern region of the state of Bahia and its refuge (dark grey) and non-refuge (light grey) areas. On the right side are the 10 fragmented landscapes with different percentages of remaining forest (grey areas) represented by the following letters: (a) 5% forest habitat, (b) 15%, (c) 20%, (d) 25%, (e) 30%, (f) 35%, (g) 40%, (h) 45%, (i) 50%, (j) 55%.

We conducted the sampling in the portion of Atlantic Forest in the state of Bahia (north-eastern Brazil) (Fig 1), a vast region between 11°80’S and 18°49’S and longitudes 21°24’W and 40°08’W. It is recognised as a biodiverse region [45] with a history of exploration dating back to the sixteenth century [46]. Selective logging and the replacement of native vegetation for livestock and agricultural production resulted in landscapes with few remnants, mostly formed by secondary or disturbed forests surrounded by a heterogeneous matrix, and dominated by pastures intermixed with crops such as cocoa, rubber, eucalyptus, banana, palm oil and coffee [47].

Studies on biogeography and phylogeography suggest that the Bahia Atlantic Forest contains centres of endemism of several taxonomic groups [48–50]. Recent studies indicate that during the late Quaternary climatic fluctuations, a large portion of the Brazilian Northeast was climatically stable, maintaining forest refuges throughout this period, which would have contributed to the maintenance of higher levels of genetic diversity in their current populations [51,52]. These studies indicate that most of the Bahia Atlantic Forest contained forest vegetation throughout the Quaternary, but its coastline vegetation is a more recent expansion.

### Landscapes sampling

We sampled dung beetles in forest and matrix habitats in 10 landscapes varying in their percentages of forest cover (5 to 55%) and in two zones with different biogeographical histories. The size of the landscape was chosen with an aim to include processes relevant to the maintenance of species of the different groups under consideration by the project, which had a maximum dispersion capacity of a few kilometres. We know very little about the dispersal capacity of dung beetles as a group or on the variation of this process among species and landscapes [44]. While it is expected that they are good dispersers, given the ephemeral resources they use for food and nesting (i.e., primarily mammalian faeces), quantitative descriptions of dung beetle dispersal are few [44]. However, the discontinuity of habitats, intensified when they are structurally very different, serves as a strong barrier to dung beetle dispersal and hence restricts their movement patterns [44].

We chose landscapes in two zones with different biogeographical histories: an interior area, which remained as a forest refuge throughout the Quaternary, and a coastal region of a recent forest expansion. We defined such zones using the MAXENT software and following the procedures described by Carnaval et al. (2008). We created three maps of forest distribution for the state of Bahia in three periods: 21,000 years ago, 6,000 years ago and the current time. This procedure generates probability values for each pixel that has been forested on the map in each period. The pixels were ranked in probability ranges: low (0 to 40%), intermediate (40 to 60%) and high (60 to 100%). We considered areas of recent forest expansion those areas that had probabilities lower than 40% on the maps of the three periods and refuge areas those that had probabilities higher than 60% on the maps of the three periods.

We overlaid the map of both zones on the current forest cover map ‘Atlas dos Remanescentes Florestais da Mata Atlântica’ (www.sosma.org.br and www.inpe.br), which was constructed through the manual classification of satellite images taken in 2008 (Landsat and CBERS). On top of them, we added one grid with 1,500 cells of 6×6 km (3,600 ha). This set of cells (defining landscapes) represented our sample universe. From the forest cover map, we calculated the proportion of forest cover in each cell (i.e. each landscape). We classified the landscapes based on the percentage of forest cover into classes of 5, 10, 15, 20, 25, 30, 35, 40, 45, 50, 55 and 60%. In each class, we included landscapes with a value of X ± 2% (e.g., landscapes with values between 3 and 7% were included in the 5% class).

We used some selection criteria before randomly choosing one landscape from each forest cover class. These criteria were intended to make the landscapes sampled more homogeneous with respect to other factors that could influence the species richness. As the level of structural similarity between the matrix and the forest affects the effect of forest loss on forest species [20], we selected only landscapes in which at least 80% of the matrix represented low-profile, anthropogenic vegetation, such as pasture fields or upland crops/shrubs. We avoided forest-like matrices, such as agroforests and tree plantations, which may minimise the effects of forest loss and fragmentation. We also controlled for the percentage of forest cover and the presence of larger forest patches in the areas surrounding the studied landscapes, as both could act as source areas for the sampled landscapes. Therefore, we established that a square of 18×18 km centred on the landscape of 6×6 km should not have a percentage of forest cover higher than that of the sampled landscape. Additionally, we established that the landscapes of 18×18 km should not have an LPI (largest patch index) [53] higher than that found in the landscapes of 6×6 km. This index represents the percentage of the landscape occupied by the largest patch. This index provides information about the dominance of the largest patch in the total area assessed, and it could be used to compare the landscapes of 6×6 km with its equivalent of 18×18 km. To calculate the LPI in the landscapes, we used the software Fragstats, version 3.3.

We interspersed the sampled areas with different forest cover values between the two zones with different biogeographic histories: the refuges and the recently expanded forests. The landscapes selected in the inland zone (refuges) were all those represented by even values of forest cover (10, 20, 30, 40, 50, and 60%). The landscapes selected in coastline zone (recently expanded forests) were those represented by odd values (5, 15, 25, 35, 45, and 55%). Finally, we visited the selected landscapes prior to the start of the study to evaluate the general quality of the forests, the non-forested matrix and the existence of roads to access the whole landscape. If the landscape did not meet these habitat composition criteria or was logistically problematic, we selected a new landscape and verified its quality.

Heavy rains has made impossible to sample in the landscapes of 10% and 60% forest cover. The sampled 10 landscapes were located in the municipalities of Ilhéus (ILH, 5% forest cover), Presidente Tancredo Neves (PTN, 15%), Itapetinga (ITA, 20%), Valença (VAL, 25%), Ubaíra (UBA, 30%), Nilo Peçanha (NPE, 35%), Wenceslau Guimarães (WGU, 40%), Camamú (CAM, 45%), Iguaí (IGU, 50%) and Jaguaripe (JAG, 55%) (Fig 1).

Each landscape was divided into 100 squares of 600×600 m, classified as either forest or matrix plots. We randomly chose eight forest and eight matrix plots to establish our sampling sites. To minimise the effects of other variables, we only sampled forests in intermediate or advanced stages of succession to control for differences in successional stage among forest patches and landscapes. We also avoided sampling in the rainy season, between May and July, and on rainy days to control for the precipitation level, and we established a minimum distance of at least 50 m from the edge of each habitat, forest and matrix, to control for the distance to the edge.

### Dung beetle sampling

We sampled the dung beetles in all 160 plots, the eight forest and matrix plots in each of the ten landscapes, using pitfall traps. In each plot, we established ten sampling stations, each with four pitfall traps every 10 m made of 500 ml clear plastic cups, 85 mm wide at the opening, 120 mm in depth, and half-filled with 90% alcohol, connected to plastic drift fences that were 1 m length and 15 cm height. The pitfall traps were active for five days (120 hours), and we checked their integrity in the middle of each sampling period. The total sampling effort was four pitfall traps/sampling station × 10 sampling stations/plot × 16 plots/landscape × 5 days, summing to 3,200 pitfall traps × 5 days/landscape.

Fernando Zagury Vaz-de-Mello and Rafael Vieira Nunes identified the dung beetles at the Scarabeoidology Laboratory of the Universidade Federal do Mato Grosso, Brazil.

### Data analysis

We classified the sampled beetle species into three habitat use categories: forest specialist, habitat generalist, and open-area specialist species. In a previous study, we observed substantial agreement between two species classification methods. One used empirical data on occurrence in the forest and/or matrix and a species indicator value analysis referred to as the IndVal index [54], considering species with at least 20 individuals recorded, while the other considered habitat use information for the same species based on the literature. Therefore, we used these concordant classification methods for species with 20 or more individuals found in our samples. We classified species with fewer than 20 individuals based on information regarding their geographical distribution in Brazilian biomes and their use of habitats presented in the literature. A total of 15 of the 17 rare species remained unclassified due to a lack of both sufficient individuals and literature descriptions of habitat associations.

For each of the three categories of habitat use, as well as for the total community and unclassified species, we calculated (1) the total number of captured individuals (abundance) and the number of species (alpha diversity) in each matrix and forest plot, and (2) the total number of captured species across all 16 plots within each landscape (gamma diversity). We calculated both alpha and gamma diversity using the observed, rather than the estimated, number of species, as the calculation of most non-parametric estimators is based on the abundance or incidence of rare species [55].

For all three categories of habitat use, we evaluated the shape of the response of abundance, alpha and gamma diversity to the percentage of forest cover at the landscape scale by fitting our data to four types of curves, that describes the possible theoretical responses for that particular group to forest loss. The responses are 1. A null model, in which there is no relationship between the dependent variable and forest cover, all data have the same expectation—the mean of the dependent variable (NULL). 2. A constant diversity decrease due to forest cover reduction, which is represented by a linear model (GLM). 3. A logistic model, which is an “S” shaped curve and represents a non-linear response with an extinction threshold model (LOG). It predicts that at higher values of forest cover the diversity levels are expected to be maintained during the process of forest reduction, and close to a critical point the diversity starts to drop exponentially to a lower value that can be zero. We used a four-parameter logistic model (see Pinheiro & Bates 2000 and Crowley 2007 [56,57]), and in a simple form it can be expressed as: y = d+ (a/ 1+exp((b-x)/c)). One parameter represents the lower value of diversity (d). Another parameter (a) represents the amount of diversity loss or the difference between the upper and the lower value. Another parameter represents the point of forest cover where the net rate of diversity change is maximum (b) that corresponds to the point where 50% of diversity loss occurred. And the last parameter represents the exponential rate of diversity change, or how fast the diversity is reduced relative to forest reduction (c). Whenever the logistic model is selected, we performed a piecewise regression to find the estimated value where the diversity starts to drop from the upper value, sometimes called the "extinction threshold", 4. The last response pattern is an inverted "U" model that represents a response in which the diversity reaches its maximum somewhere during the habitat loss process, more commonly associated with generalists species. We chose the general bell-shaped curve, y = a. exp(|b.(x-d)|^2^)+c (BELL).

For the stochastic part of the model, we chose distribution families that are conceptually more adequate to each type of data. Abundance was modeled using Poison distribution. Mean alfa and gamma diversity were modeled using normal distribution, and we tested for normality of residuals of the fitted models and overdispersion in the case of Poison errors. We fitted the data to the models by building a numerical optimization function [58] for each model. These functions return the best values for the models coefficients as well as the likelihood value of the model, prone to use in a multimodel selection.

Finally, we performed a model selection [59], and we used corrected AIC values to find the best models, and in case that we find more than one best model (delta AIC < = 2), we also used further analysis to select among them. First, we tested for insufficiency of the model (if its residuals were correlated with the independent variable). Then we discuss the consequences of admitting each of the best models as the shape of biological response to habitat loss.

All analyses were conducted in the R environment [60] using the packages ‘stats’, ‘segmented’ [61], and ‘bbmle’ [62].

## Results

We recorded 7,673 individuals of 42 dung beetle species in the 10 sampled landscapes (Table 1) and excluded 20 species from further analysis due to insufficient information to classify their habitat use. Although the species removed from analysis represent 48% of the species sampled in the study, they represent less than 1% of the sampled individuals.

**Table 1:** Species classification into habitat use categories.

Species	N	Habitat use category		Final habitat use classification
		According to the literature	According to the empirical data	
*Ateuchus oblongus*	2,553	FS	FS	FS
*Canthidium punctatostriatum*	30	II	FS	FS
*Canthidium sp*. *7*	28	II	G	G
*Canthon curvodilatatus*	56	OS	OS	OS
*Canthon nigripennis*	7	G	II	G
*Canthon septemmaculatus histrio*	4	G	II	G
*Canthon staigi*	36	G	G	G
*Canthonella barrerai*	43	FS	FS	FS
*Coprophanaeus acrisius*	13	G	II	G
*Coprophanaeus dardanus*	11	FS	II	FS
*Deltochilum brasiliense*	12	FS	II	FS
*Deltochilum calcaratum*	48	FS	FS	FS
*Dichotomius ascanius*	2	G	II	G
*Dichotomius bos*	2	G	II	G
*Dichotomius nisus*	11	G	II	G
*Dichotomius semisquamosus*	10	G	II	G
*Dichotomius* sp. aff. *fissus*	355	II	FS	FS
*Dichotomius* sp. aff. *sericeus*	4,357	II	FS	FS
*Digitonthophagus gazella*	7	OS	II	OS
*Ontherus appendiculatus*	21	G	G	G
*Trichillum externepunctatum*	4	G	II	G

N, number of individuals collected; FS, forest specialist species; II, insufficient information; G, habitat generalist species; OS, open-area specialist species.

**Fig 2:**
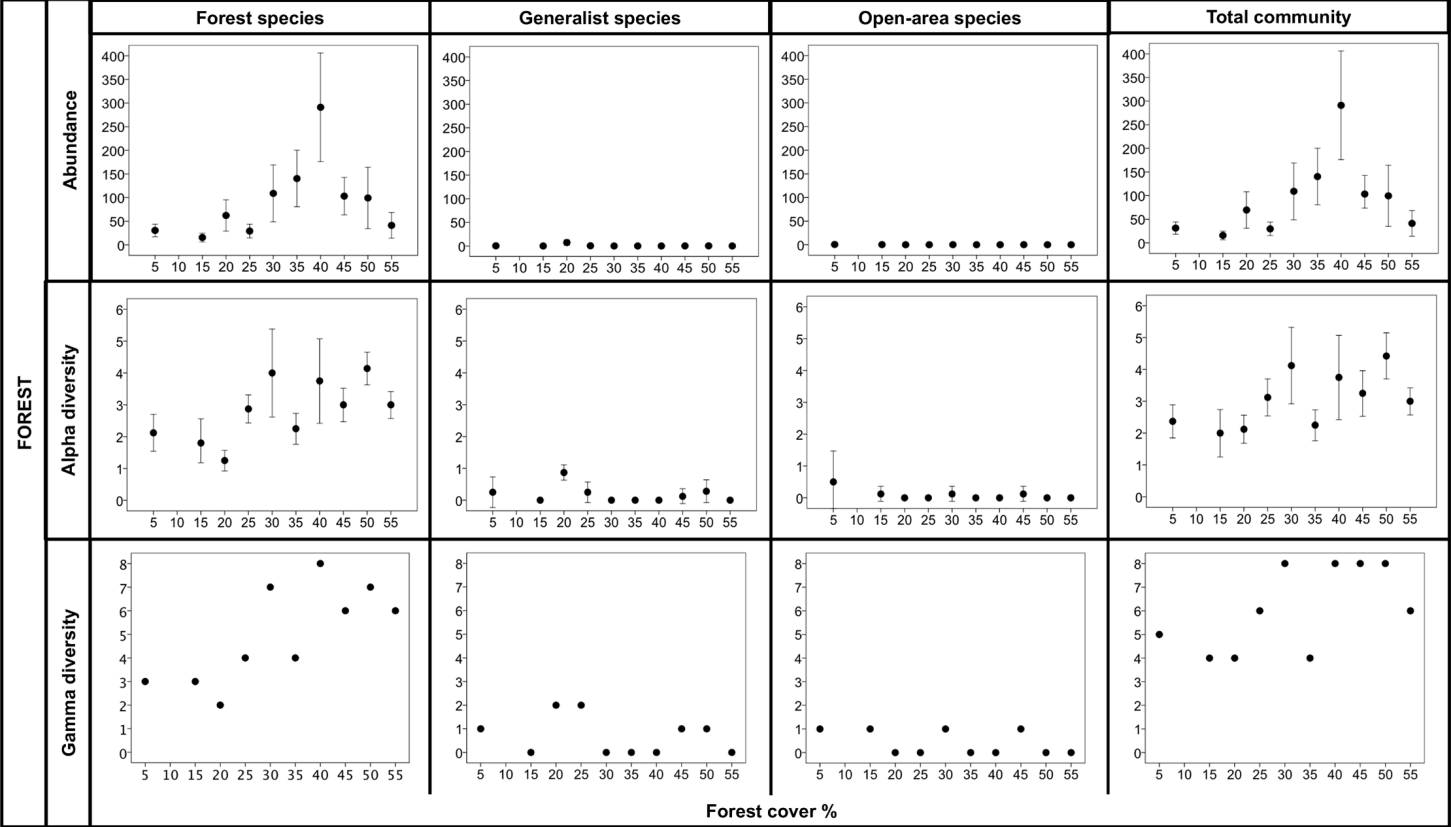
Abundance, alpha diversity, and gamma diversity in forests across landscapes with different amount of forest cover. For the abundance and alpha diversity, the mean and 95% confidence intervals among for the eight surveyed plots per landscapes are shown.

In the forest plots, forest specialist species responded to the forest cover gradient at the landscape scale (Fig 2). While the alpha diversity and gamma diversity of the forest species declined similarly as a function of forest loss, their abundance behaved differently with the forest cover gradient. There was an increase in abundance at approximately 30% forest cover, which was maintained until the landscape had 50% coverage, later declining in the landscape with 55% forest cover. The peak in abundance observed in the landscape with 40% forest cover was related to the strong contribution of individuals of a single collected species, *Dichotomius* sp. aff. *sericeus*, with 2,221 individuals.

**Fig 3:**
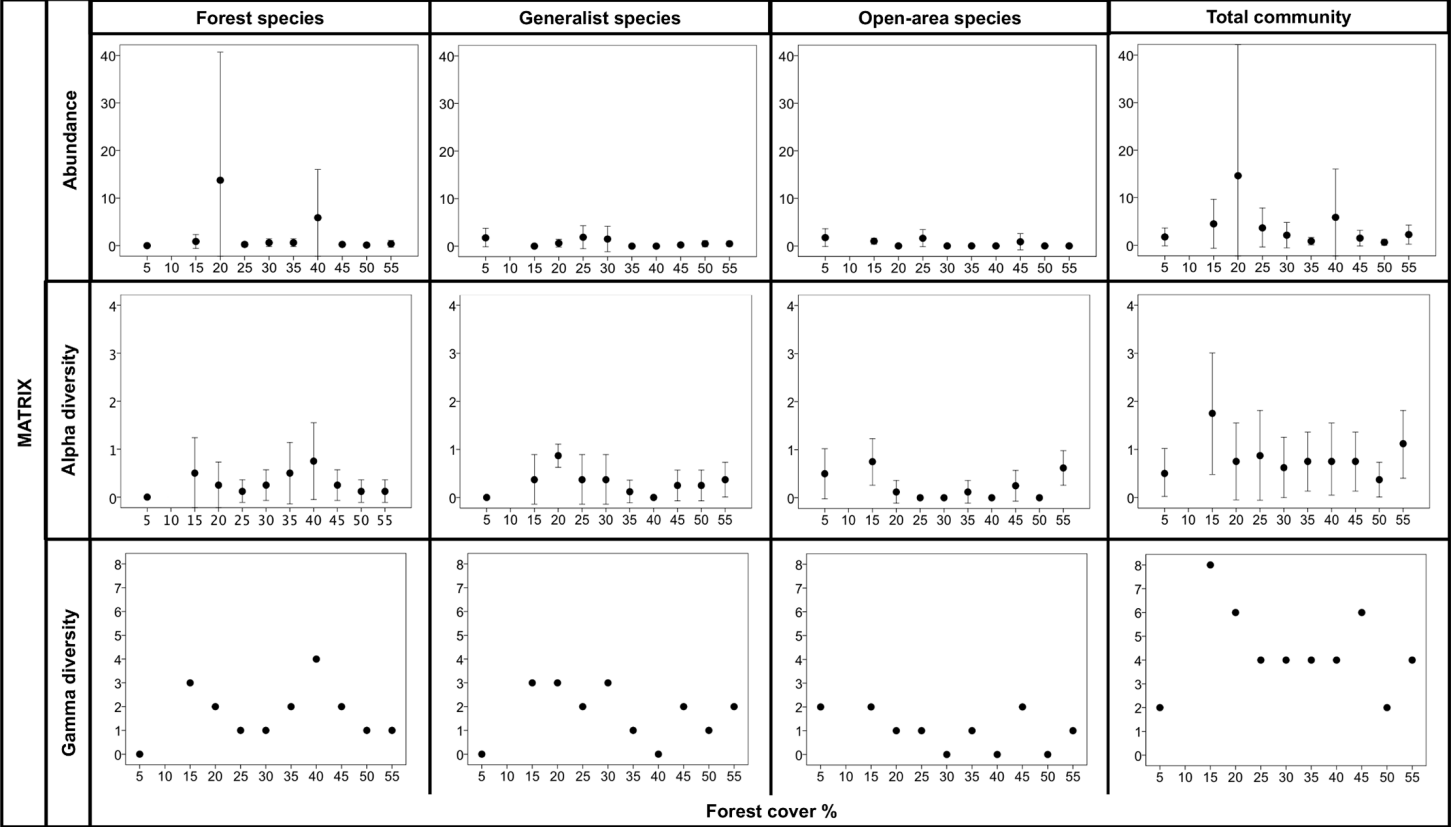
Abundance, alpha diversity, and gamma diversity in matrix habitat across landscapes with different amount of forest cover. For the abundance and alpha diversity, the mean and 95% confidence intervals for the eight surveyed plots per landscape are shown.

We recorded very low densities of forest specialist species in the matrix plots throughout all the landscapes (Fig 3), except in one of the eight matrix plots in the landscape with 20% forest cover, in which we recorded 107 individuals of *Dichotomius* sp. aff. *sericeus*. Similarly, in the landscape with 40% forest cover, we found 40 individuals of the same species in only one matrix plot. The alpha diversity of forest specialists in the matrix plots was also low across the forest cover gradient, with one or less than one species on average per plot in each landscape. In the same way, we recorded low values of gamma diversity in the matrix plots, varying between 0 and 4 species independent of the forest cover in the landscape.

**Fig 4:**
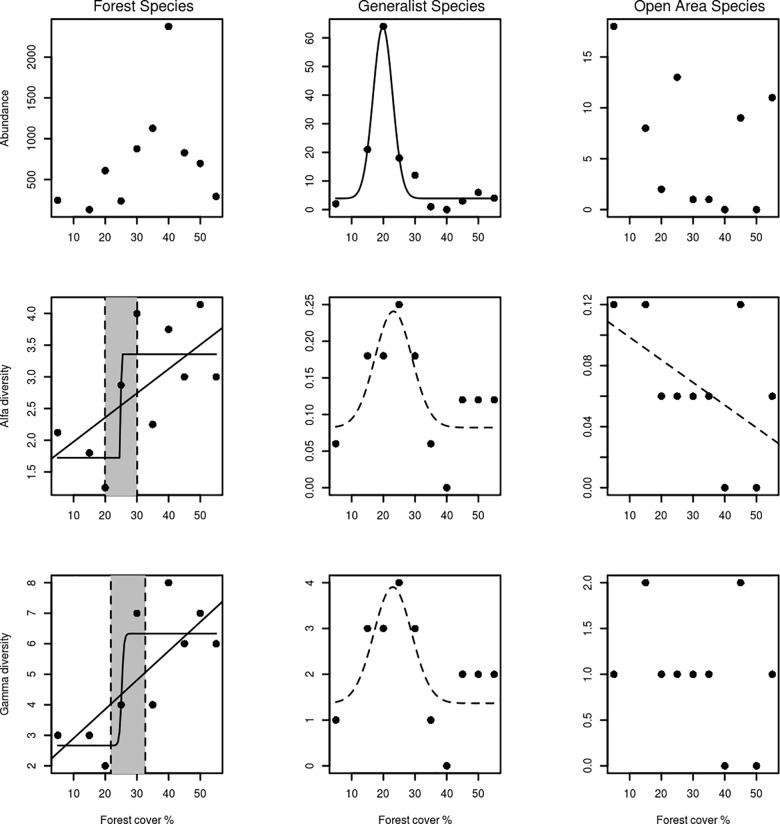
Abundance, alpha diversity and gamma diversity of dung beetle groups across landscapes with different amount of forest cover. Solid lines correspond to the best-fitted models. Dashed lines correspond to models that fit equally to the null model. The grey area corresponds to the confidence interval around the threshold value.

The fit of the pattern for forest species alpha diversity presented similar delta AICc values of GLM, LOG and NULL in the model selection procedure (Table 2). Since the logistic model was one of the best models, we calculated the associated threshold value and its confidence interval as 24.7 ± 5.1% forest cover (Table 2, Fig 4). The fit of the pattern for forest species gamma diversity presented similar delta AICc values for the GLM and LOG (Table 2). The threshold (± its confidence interval) was estimated as occurring at 25.2 ± 7.4% forest cover (Table 2, Fig 4). We found a significant and positive correlation between the residuals of the NULL and the forest cover (p = 0.04). This result indicates that there is a pattern in the residuals, and therefore this model is insufficient to fit the data. The residuals of GLM and LOG did not present this pattern in association with the residuals.

**Table 2:** Best models (in bold) for explaining the relationships between diversity measures of dung beetle groups and the forest amount at the landscape scale.

Species group	Diversity model	ΔAICc	K	wi	Abundance model	ΔAICc	K	wi
Forest species	Alpha							
	**GLM**	**0.0**	**3**	**0.45**	**NULL**	**0.0**	**2**	**0.82**
	**NULL**	**0.9**	**2**	**0.29**	GLM	3.0	3	0.18
	**LOG**	**1.1**	**4**	**0.26**	LOG	2541.4	4	<0.001
	Gamma							
	**GLM**	**0.0**	**3**	**0.54**				
	**LOG**	**0.7**	**4**	**0.38**				
	NULL	3.7	2	0.08				
Generalist species	Alpha							
	**NULL**	**0.0**	**2**	**0.46**	**BELL**	**0.0**	**4**	**1**
	**BELL**	**0.0**	**4**	**0.46**	NULL	25.4	2	<0.001
	GLM	3.8	3	0.07	GLM	28.0	3	<0.001
	LOG	7.8	4	0.01	LOG	74.7	4	<0.001
	Gamma							
	**NULL**	**0.0**	**2**	**0.49**				
	**BELL**	**0.3**	**4**	**0.41**				
	GLM	3.8	3	0.07				
	LOG	5.6	4	0.03				
Open-area species	Alpha							
	**NULL**	**0.0**	**2**	0.58	**NULL**	**0.0**	**2**	**0.78**
	**GLM**	**0.9**	**3**	0.37	GLM	2.6	3	0.22
	LOG	4.9	4	0.05	LOG	22.3	4	<0.001
	Gamma							
	**NULL**	**0.0**	**2**	**0.83**				
	GLM	3.3	3	0.16				
	LOG	9.0	4	0.01				

The fit of the pattern for generalist species abundance presented higher delta AICc values for BELL (Table 2); a peak in abundance was found in landscapes with 20 to 25% forest cover (Fig 4). Alpha and gamma diversity also presented bell-shaped responses, but in such cases, these responses were as equally probable as the null model. This pattern of a clear response in abundance associated with weaker response of diversity resulted from a very species-poor generalist community, primarily composed of three species, *Canthon nigripennis* (recorded in landscapes with 25% forest cover), *Canthon staigi* (recorded in landscape with 20% forest cover) and *Canthidium* sp. 7 (recorded in landscapes with 20% and 25% forest cover) (Fig 2).

The open-area specialist species also have a very species-poor community, similar to the habitat generalist species, however with lower values of abundance recorded. Their response to the forest cover gradient showed no pattern at all, as they were non-existent in forests, and all of the few specimens recorded belong to the same species, *Canthon curvodilatatus*. Null models were selected for the total community and for unclassified species.

## Discussion

Our results suggest that: (1) a reduction in forest cover at the landscape scale modifies the dung beetle community subgroups in different ways; (2) forest specialist species respond to the forest cover gradient in a way that fits both linear and threshold models: if threshold is assumed, an abrupt decline in their diversity occurs when forest cover reduces beyond the limit of 25% of the landscape; (3) the response of forest specialist dung beetles species to the reduction of forest cover in the landscape was similar to that of other taxonomic groups, such as vertebrates and plants; (4) habitat generalist species, despite their lower richness, responded to the forest cover gradient at the landscape scale when we considered their abundance, showing a peak at approximately 20% forest cover; and (5) open-area specialists are a very rare group in the Atlantic Forest of Bahia, and their species appear to be less influenced by forest cover in the landscapes.

One of our probable results showed that forest specialist dung beetles declined abruptly in landscapes with less than 25% of remaining forests, similarly to other taxonomic groups, such as vertebrates and plants [5–7,9,11,22]. The mechanism that explains the steep reduction in species with the loss of forest cover in the landscape is based mainly on the exponential increase in distances among patches that would occur around ~20% forest cover associated with a decrease in forest patch number and an increase in edge density [16]. There are remarkable differences in structure and microclimatic factors between the two habitats sampled in our study, pastures and forests. In addition, the degree of habitat specialisation of forest specialist dung beetle has already been noted [39–41]; therefore, it was not surprising that their subpopulations were subject to the effects of the increase in distance among patches along with a reduction in forest cover in the landscape.

Dung beetles responded to the decrease in forest cover in a similar way than to that of larger organisms, even considering their supposed larger minimum area requirement. However, some authors observed in an archipelago created by floods in Venezuela that the most fragmentation-sensitive dung beetles appeared to require fragments even larger than 85 ha, an area that was sufficient to maintain many larger vertebrates [37]. In the same study, forest specificity was one of the three traits of extinction-prone dung beetle species. The other two were large body size and low population density. In addition, the mechanism that may contribute to the local extinction of forest specialists was their poor dispersal capacity across the landscape matrix, which may decrease their persistence by constraining immigration.

In more deforested landscapes (i.e., forest cover below 40%), the forest remnants present structural degradation through the influence of edge effects [13]. In addition, this may be reflected in the poor quality of soil and microclimatic conditions in these remnants, which could be big enough to maintain viable populations of dung beetles but have very different quality for maintaining viable populations of sensitive species of dung beetles. Even considering that dung beetles have diverse feeding habits, such as necrophagy, saprophagy, and coprophagy, including the use of human faeces [27], the loss of small and medium-sized mammals in landscapes with low forest cover (below 40% and 30%, respectively) certainly decreases the amount and diversity of food and nesting resources and contributes to the adverse effects on this group.

Despite the fact that the model selection analysis did not support only one model as clearly being best for the diversity analysis, it is not unusual to find data that do not support only one model [59]. According to Anderson, the frequency of such inability is not a defect of AICc or any other selection criterion; rather, it is an indication that the data do not support strong inferences [59]. However, AICc values are functions of sample size and model complexity (e.g., nonlinear models), and more data should probably be collected to improve their ranking [63].

The results of the present study do not allow to statistically discriminate between the fit of the data obtained for both models (linear and nonlinear) of forest species loss with forest loss at the landscape scale. However, the threshold model represents a more dramatic scenario for the loss of biodiversity, since a small reduction in forest cover (from 30% to 20% of forest cover in the landscape) leads to reductions of species richness in the plot (49%) and in the landscape (56%) similar to those predicted by the linear model along a larger reduction of forest cover (from 50% to 5% of forest cover in the landscape), that is, 53% in the plot and 70% in the landscape. We suggest, therefore, based on the precautionary criterion, that in establishing guidelines for the management of the minimum amount of habitat in the landscape aiming at biodiversity conservation, our results should be interpreted assuming the biodiversity threshold model. Other communities studied in the same project (e.g., plants, birds, and small mammals) [6,7,9] and another region of Atlantic Forest (e.g., small mammals and birds) [5,8] responded in a nonlinear way to the reduction of forest cover in the landscape, indicating a biodiversity threshold varying from 50% to 30% of forest cover. Therefore it would be precautious to avoid reducing the amount of Atlantic Forest in landscapes beyond 50% if the conservation of small to medium-sized species of animals and zoochoric plant species with small to medium-sized seeds is intended. Larger birds, larger mammals and plants that depend on them for seed dispersal are known to be even more sensitive to forest loss and pervasive synergistic effect of other anthropogenic disturbances [12,64].

Regarding generalist species, our results were similar to those observed for generalist small mammals [6] and birds [10], which also showed a peak in abundance, but at 30% in 50% forest cover, respectively. However, the peak in abundance that we observed was associated with a very species-poor generalist community, and with a very lower quantity when compared with the abundance values of forest species. Therefore, we did not observe a compensatory effect of habitat generalist species with the local extirpation of forest-dependent species as observed with dung beetle community in Pernambuco Atlantic rainforest [41]. In our results, habitat generalists occurred both in the matrix habitat and in the forest in the landscapes, with low and constant species richness and species abundance along the gradient.

Finally, we did not observe any response of open-area specialists in terms of the variation in the amount of forest. They were the less diverse subgroup and also occur at very low abundance and richness across the gradient of forest cover, even in matrix habitats. Low abundance in the matrix was evident for all three categories of habitat use. This result resembles what was found in other studies in which traps set in open habitats within forested landscapes typically catch very few species [33,34,65]. The low abundance of dung beetles found in the matrix of all landscapes, even those with higher forest cover, suggest that in the studied Atlantic rainforest landscapes, this group has a significant dependence on forested habitats and that it is in these environments that we can expect pronounced positive effects on ecosystem functioning. In these environments, dung beetles play an important role in maintaining ecosystem integrity, in particular through secondary seed dispersal and nutrient cycling [30]. If we want to maintain diverse and functional forests, it is imperative to understand and protect this biodiversity and these processes.

Despite the highly threatened status of the Atlantic Forest, its continued maintenance of high levels of diversity and endemism [66] make it one of the world’s biodiversity hotspots [67]. Unfortunately, the level of threat to this particular biome has not decreased in recent years [68]. Deforestation and defaunation in the Atlantic Forest have a long-documented history, and if current trends carry on in the region, the diversity of this group of insects as well as vertebrates and plants will be severely threatened. Pardini and others observed a possible regime shift in the Atlantic Forest landscapes, where the loss of most specialist species and the proliferation of generalist species would occur [5]. For the dung beetles sampled in this study, the situation is worse: landscapes with less than 30% of the vegetation remaining demonstrate a sudden loss of the forest specialist species without an increase in generalist species or open-area specialists. Dung beetle communities that occupy landscapes with low forest cover are highly impoverished. Therefore, we support the urgency of conservation initiatives that analyse and protect larger amounts of forest cover in the landscapes, which restore deforested areas to improve connectivity among patches and enhance matrix quality to avoid structural and microclimatic discrepancies between habitats. Only then will we be taking the first steps towards a prosperous future for Brazilian Atlantic Forest.

## Supporting information

S1 Table Dung beetles (Coleoptera: Scarabaeinae) collected in ten Atlantic forest landscapes with forest cover ranging from 5% to 55%. (DOCX)
