# Supplementary material for: Biodiversity thresholds in invertebrate communities: The responses of dung beetle subgroups to forest loss
Source: PLoS One. 2018 Aug 10;13(8):e0201368. doi: 10.1371/journal.pone.0201368 (PMC6086426; doi:10.1371/journal.pone.0201368)
Supplement: S1 Table — (DOCX) [file pone.0201368.s001.docx]

**S1 Table. Dung beetles (Coleoptera: Scarabaeinae) collected in ten Atlantic Forest landscapes with forest cover ranging from 5% to 55%.** M, matrix plots; F, forest plots.

| **Species** | **Number of individuals collected per habitat per landscape** | | | | | | | | | | | | | | | | | | | |
| --- | --- | --- | --- | --- | --- | --- | --- | --- | --- | --- | --- | --- | --- | --- | --- | --- | --- | --- | --- | --- |
|  | **5**  **M** | **5**  **F** | **15**  **M** | **15**  **F** | **20**  **M** | **20**  **F** | **25**  **M** | **25**  **F** | **30**  **M** | **30**  **F** | **35**  **M** | **35**  **F** | **40**  **M** | **40**  **F** | **45**  **M** | **45**  **F** | **50**  **M** | **50**  **F** | **55**  **M** | **55**  **F** |
| *Anomiopus* sp1 | 0 | 1 | 0 | 0 | 0 | 0 | 0 | 0 | 1 | 0 | 0 | 0 | 0 | 0 | 0 | 0 | 0 | 0 | 0 | 0 |
| *Ateuchus asperatus* | 0 | 2 | 0 | 0 | 0 | 0 | 0 | 0 | 0 | 0 | 0 | 0 | 0 | 0 | 0 | 0 | 0 | 0 | 0 | 0 |
| *Ateuchus oblongus* | 0 | 143 | 4 | 21 | 0 | 0 | 2 | 113 | 5 | 678 | 2 | 806 | 3 | 117 | 1 | 368 | 0 | 182 | 0 | 87 |
| *Ateuchus* sp2 | 0 | 1 | 0 | 0 | 0 | 0 | 0 | 0 | 0 | 0 | 0 | 0 | 0 | 0 | 0 | 0 | 0 | 0 | 0 | 0 |
| *Canthidium punctatostriatum* | 0 | 0 | 0 | 0 | 3 | 7 | 0 | 0 | 0 | 5 | 0 | 1 | 0 | 5 | 0 | 3 | 0 | 2 | 0 | 3 |
| *Canthidium haagi* | 0 | 0 | 0 | 0 | 0 | 0 | 0 | 1 | 0 | 1 | 0 | 0 | 0 | 0 | 0 | 0 | 0 | 0 | 0 | 0 |
| *Canthidium* sp5 | 0 | 0 | 0 | 0 | 0 | 0 | 0 | 1 | 0 | 0 | 0 | 0 | 0 | 0 | 0 | 0 | 0 | 0 | 0 | 2 |
| *Canthidium* sp7 | 0 | 0 | 0 | 0 | 3 | 24 | 0 | 1 | 0 | 0 | 0 | 0 | 0 | 0 | 0 | 0 | 0 | 0 | 0 | 0 |
| *Canthidium* sp8 | 0 | 0 | 0 | 0 | 0 | 0 | 0 | 0 | 0 | 0 | 0 | 0 | 0 | 0 | 0 | 0 | 0 | 0 | 0 | 1 |
| *Canthidium* sp9 | 0 | 0 | 0 | 0 | 0 | 0 | 0 | 0 | 0 | 0 | 0 | 0 | 0 | 0 | 0 | 0 | 0 | 0 | 0 | 2 |
| *Canthidium* sp10 | 0 | 0 | 0 | 0 | 0 | 0 | 0 | 0 | 0 | 0 | 0 | 0 | 0 | 1 | 0 | 0 | 0 | 0 | 0 | 0 |
| *Canthon curvodilatatus* | 12 | 4 | 6 | 2 | 0 | 0 | 13 | 0 | 0 | 1 | 1 | 0 | 0 | 0 | 7 | 1 | 0 | 0 | 11 | 0 |
| *Canthon nigripennis* | 0 | 2 | 0 | 0 | 0 | 0 | 0 | 2 | 1 | 0 | 0 | 0 | 0 | 0 | 1 | 1 | 0 | 0 | 0 | 0 |
| *Canthon staigi* | 0 | 0 | 0 | 0 | 35 | 1 | 0 | 0 | 0 | 0 | 0 | 0 | 0 | 0 | 0 | 0 | 0 | 0 | 0 | 0 |
| *Canthon septemmaculatus histrio* | 0 | 0 | 0 | 0 | 0 | 0 | 2 | 0 | 0 | 0 | 1 | 0 | 0 | 0 | 0 | 0 | 0 | 0 | 0 | 0 |
| *Canthonella barreirai* | 0 | 7 | 2 | 3 | 0 | 0 | 0 | 8 | 0 | 2 | 0 | 0 | 0 | 1 | 0 | 4 | 0 | 6 | 0 | 11 |
| *Coprophanaeus acrisius* | 0 | 0 | 0 | 0 | 0 | 0 | 13 | 0 | 0 | 0 | 0 | 0 | 0 | 0 | 0 | 0 | 0 | 0 | 0 | 0 |
| *Coprophanaeus dardanus* | 0 | 0 | 0 | 0 | 0 | 0 | 0 | 0 | 0 | 0 | 0 | 0 | 0 | 3 | 0 | 1 | 0 | 0 | 0 | 7 |
| *Dendropaemon* sp. | 0 | 0 | 0 | 0 | 0 | 0 | 1 | 0 | 0 | 0 | 0 | 0 | 0 | 0 | 0 | 0 | 0 | 0 | 0 | 0 |
| *Deltochilum brasiliense* | 0 | 0 | 0 | 0 | 0 | 0 | 0 | 0 | 0 | 1 | 0 | 0 | 2 | 7 | 0 | 0 | 0 | 2 | 0 | 0 |
| *Deltochilum calcaratum* | 0 | 0 | 0 | 0 | 0 | 0 | 0 | 3 | 0 | 20 | 3 | 1 | 1 | 12 | 1 | 1 | 1 | 3 | 0 | 0 |
| *Deltochilum granulosum* | 0 | 0 | 0 | 0 | 0 | 0 | 0 | 0 | 0 | 0 | 0 | 0 | 2 | 0 | 0 | 0 | 0 | 0 | 0 | 0 |
| *Dichotomius ascanius* | 0 | 0 | 0 | 0 | 0 | 0 | 0 | 0 | 0 | 0 | 0 | 0 | 0 | 0 | 0 | 0 | 0 | 2 | 0 | 0 |
| *Dichotomius bos* | 0 | 0 | 0 | 0 | 0 | 0 | 0 | 0 | 1 | 0 | 0 | 0 | 0 | 0 | 1 | 0 | 0 | 0 | 0 | 0 |
| *Dichotomius geminatus* | 0 | 0 | 0 | 0 | 0 | 0 | 0 | 0 | 0 | 0 | 0 | 0 | 0 | 0 | 0 | 0 | 0 | 0 | 2 | 0 |
| *Dichotomius nisus* | 0 | 0 | 1 | 0 | 1 | 0 | 0 | 0 | 7 | 0 | 0 | 0 | 0 | 0 | 0 | 0 | 0 | 0 | 2 | 0 |
| *Dichotomius semisquamosus* | 0 | 0 | 0 | 0 | 0 | 0 | 0 | 0 | 4 | 0 | 0 | 0 | 0 | 0 | 0 | 0 | 4 | 0 | 2 | 0 |
| *Dichotomius socius* | 0 | 0 | 0 | 0 | 0 | 0 | 0 | 0 | 0 | 4 | 0 | 0 | 0 | 0 | 0 | 0 | 0 | 0 | 0 | 0 |
| *Dichotomius* aff. *sericeus* | 0 | 95 | 1 | 101 | 107 | 491 | 0 | 110 | 0 | 116 | 0 | 315 | 41 | 2180 | 0 | 448 | 0 | 201 | 3 | 179 |
| *Dichotomius* aff. *fissus* | 0 | 0 | 0 | 0 | 0 | 0 | 0 | 0 | 0 | 50 | 0 | 0 | 0 | 4 | 0 | 0 | 0 | 299 | 0 | 2 |
| *Digitonthophagus gazella* | 2 | 0 | 2 | 0 | 2 | 0 | 0 | 0 | 0 | 0 | 0 | 0 | 0 | 0 | 1 | 0 | 0 | 0 | 0 | 0 |
| *Eurysternus nanus* | 0 | 0 | 1 | 0 | 0 | 0 | 0 | 1 | 0 | 0 | 0 | 0 | 0 | 0 | 0 | 1 | 0 | 0 | 0 | 0 |
| *Holocephalus sculptus* | 0 | 0 | 0 | 0 | 0 | 0 | 0 | 0 | 0 | 0 | 0 | 0 | 0 | 1 | 0 | 0 | 0 | 0 | 0 | 0 |
| *Ontherus appendiculatus* | 0 | 0 | 19 | 0 | 0 | 0 | 0 | 0 | 0 | 0 | 0 | 0 | 0 | 1 | 0 | 0 | 0 | 0 | 0 | 0 |
| *Ontherus zikani* | 0 | 0 | 0 | 0 | 0 | 0 | 0 | 0 | 3 | 8 | 0 | 0 | 0 | 1 | 0 | 0 | 0 | 1 | 0 | 0 |
| *Onthophagus rannuculus* | 2 | 0 | 0 | 0 | 0 | 0 | 0 | 0 | 1 | 0 | 0 | 0 | 0 | 0 | 0 | 0 | 0 | 0 | 0 | 0 |
| *Pseudocanthon* sp | 0 | 0 | 0 | 0 | 0 | 0 | 2 | 0 | 0 | 1 | 1 | 0 | 0 | 0 | 1 | 0 | 0 | 0 | 0 | 0 |
| *Scatonomus fasciculatum* | 0 | 0 | 0 | 0 | 0 | 0 | 0 | 0 | 0 | 0 | 0 | 0 | 0 | 0 | 0 | 2 | 0 | 3 | 0 | 0 |
| *Trichilum externepunctatum* | 0 | 0 | 1 | 0 | 0 | 0 | 0 | 0 | 0 | 0 | 0 | 0 | 0 | 0 | 2 | 0 | 0 | 0 | 1 | 0 |
| *Uroxys* sp. | 0 | 0 | 0 | 0 | 0 | 0 | 0 | 0 | 0 | 0 | 0 | 0 | 0 | 0 | 2 | 0 | 0 | 0 | 0 | 0 |
